# Clinical and genetic analysis of Majeed syndrome caused by LPIN2 complex heterozygous mutation and literature review

**DOI:** 10.3389/fped.2025.1602509

**Published:** 2025-10-02

**Authors:** Shasha Wang, Ting Yu, Xuelian He, Yan Ding, Yali Wu

**Affiliations:** ^1^ Department of Rheumatology and Immunology, Wuhan Children's Hospital (Wuhan Maternal and Child Healthcare Hospital), Tongji Medical College, Huazhong University of Science & Technology, Wuhan, China; ^2^ Precision Medical Center, Wuhan Children's Hospital (Wuhan Maternal and Child Healthcare Hospital), Tongji Medical College, Huazhong University of Science & Technology, Wuhan, China

**Keywords:** Majeed syndrome, LPIN2 gene, chronic recurrent multifocal osteomyelitis, compound heterozygous mutation, whole-exome sequencing

## Abstract

**Background:**

Majeed syndrome is a rare autosomal recessive autoinflammatory disorder caused by LPIN2 mutations. It is characterized by chronic recurrent multifocal osteomyelitis (CRMO), congenital dyserythropoietic anemia (CDA), and, in some cases, neutrophilic dermatoses. Its rarity and overlap with juvenile idiopathic arthritis (JIA) often lead to delayed or incorrect diagnoses.

**Case presentation:**

We report a 3-year-10-month-old girl with recurrent swelling and pain of the knees and ankles, associated with low-grade fever and elevated inflammatory markers for over two years. Initially diagnosed and treated as JIA with NSAIDs, methotrexate, and adalimumab, she experienced only partial improvement. MRI revealed multifocal bone marrow edema consistent with CRMO, and laboratory results demonstrated mild microcytic anemia. These findings raised suspicion of a monogenic autoinflammatory disease. Whole-exome sequencing identified two novel LPIN2 variants: c.2349del (p.Glu784ArgfsTer8), inherited maternally, and c.2327+3A>G, inherited paternally. RNA analysis confirmed exon 17 skipping, carried out quantitative RT-PCR analysis of LPIN2 mRNA,establishing pathogenicity of the splice-site variant. Together with the clinical features, these findings confirmed the diagnosis of Majeed syndrome. A review of 35 previously reported patients demonstrated that most presented before age three with CRMO and recurrent fever, but the severity of CDA varied widely. IL-1 blockade remains the most effective treatment, with sustained remission reported in multiple cases.

**Conclusion:**

This case expands the mutational spectrum of LPIN2 and emphasizes the importance of early genetic testing in children with recurrent osteomyelitis and anemia refractory to standard therapy. Prompt recognition enables accurate diagnosis and timely initiation of IL-1–targeted therapy, which can markedly improve outcomes.

## Background

Autoinflammatory diseases are a group of disorders characterized by recurrent episodes of systemic inflammation, caused by dysregulation of the innate immune system in the absence of high-titer autoantibodies or antigen-specific T cells (1). Majeed syndrome is a rare, monogenic autoinflammatory condition with an autosomal recessive inheritance pattern, first described in 1989, and it is clinically defined by a characteristic triad consisting of chronic recurrent multifocal osteomyelitis (CRMO), congenital dyserythropoietic anemia (CDA), and, less frequently, neutrophilic dermatoses such as Sweet syndrome (2). The disease results from pathogenic variants in the LPIN2 gene, which encodes the protein Lipin-2.

Lipin-2 has been shown to regulate activation of the NLRP3 inflammasome and the subsequent production of interleukin-1β (IL-1β), a key pro-inflammatory cytokine (3). Mutations in LPIN2 impair this regulatory function, leading to excessive IL-1β signaling and the systemic inflammation that characterizes Majeed syndrome (4). This mechanistic insight has transformed therapeutic strategies, with IL-1 antagonists now considered the most effective treatment option.

Despite these advances, significant knowledge gaps remain regarding the full clinical and genetic spectrum of Majeed syndrome. Its rarity and clinical overlap with more common pediatric rheumatic diseases, particularly juvenile idiopathic arthritis (JIA) (5), often result in delayed diagnosis and initial misclassification. Such delays can lead to prolonged exposure to less effective therapies and unnecessary patient suffering. Thus, reporting new cases, particularly those involving novel genetic variants, is essential to expand genotype–phenotype correlations and enhance diagnostic recognition.

Here, we present the case of a 3-year-old girl with Majeed syndrome caused by a previously unreported compound heterozygous mutation in LPIN2. By detailing her clinical course from initial misdiagnosis to genetically confirmed disease, this report indicates the critical role of early whole-exome sequencing in distinguishing Majeed syndrome from clinical mimics and enabling timely, targeted treatment.

## Case presentation

A 3-year and 10-month-old girl was admitted to our hospital on November 9, 2021, with a history of intermittent joint pain for more than two years, first manifesting at 1 year and 8 months of age. The episodes were characterized by recurrent swelling and pain of the knees and ankles, accompanied by limping and occasional redness, occurring four to five times annually. Each attack was associated with low-grade fever and elevated inflammatory markers, including C-reactive protein (CRP) and erythrocyte sedimentation rate (ESR). Ibuprofen provided only partial relief, and there was no history of rash, oral ulcers, cough, vomiting, or diarrhea. The child was the first pregnancy and first delivery (G1P1), and no family history of similar symptoms was reported.

On admission, her height was 96 cm and her weight was 12.5 kg. Physical examination revealed swelling, tenderness, and pain in both knees and ankles, with a positive FABER (Patrick's) test, but without erythema or involvement of other joints. Given the chronic and recurrent arthritis-like symptoms, the initial working diagnosis was juvenile idiopathic arthritis (JIA), and she was treated sequentially with naproxen, methotrexate, and adalimumab. These therapies reduced the frequency of flares but did not completely control joint pain, raising doubts about the initial diagnosis and prompting further evaluation.

During the attack phase, laboratory findings showed mild anemia (hemoglobin 103 g/L; reference 112–149 g/L), with microcytic indices (MCV 81.5 fL, MCH 26.2 pg). Inflammatory markers were significantly elevated, with hypersensitive CRP at 79.1 mg/L (reference 0–3 mg/L) and ESR at 51 mm/h (reference 0–20 mm/h). Pro-inflammatory cytokines were markedly increased, including IL-6 at 38.18 pg/ml (reference 0–20.9 pg/ml), TNF-α at 6.44 pg/ml (reference 0–5.5 pg/ml), and IL-17A at 40.64 pg/ml (reference 1–5 pg/ml). In contrast, leukocyte count, procalcitonin, and ferritin remained within normal ranges. During remission, inflammatory markers decreased (hs-CRP 7.99 mg/L, ESR 23 mm/h). Biochemistry, including liver and kidney function, electrolytes, lipids, and myocardial enzymes, were normal, but fibrinogen was elevated (6.02 g/L; reference 1.92–4.01 g/L). Immunological markers, including ANA, HLA-B27, rheumatoid factor, and anti-CCP, were negative, and 25-hydroxyvitamin D was normal.

**Figure 1:**
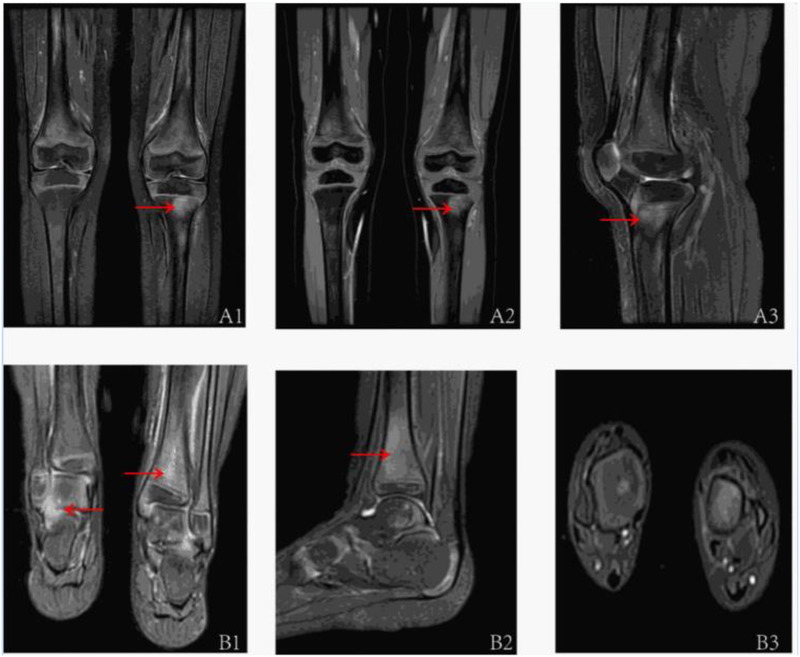
The MRI assessments of the children's joints are as follows: A1–A3 pertain to the MRI evaluations of both knee joints (A1: anterior; A2: anterior; A3: left lateral). The T2IDEAL WATER images revealed bone marrow edema in both distal femurs and the proximal left tibia. B1–B3 correspond to the MRI evaluations of both ankle joints of the patient (B1: Posterior; B2: Left Lateral; B3: Sagittal Plane), demonstrating abnormal bone signals at the distal tibia and swelling of the surrounding soft tissues, as observed in the T2IDEAL WATER images.

**Figure 2:**
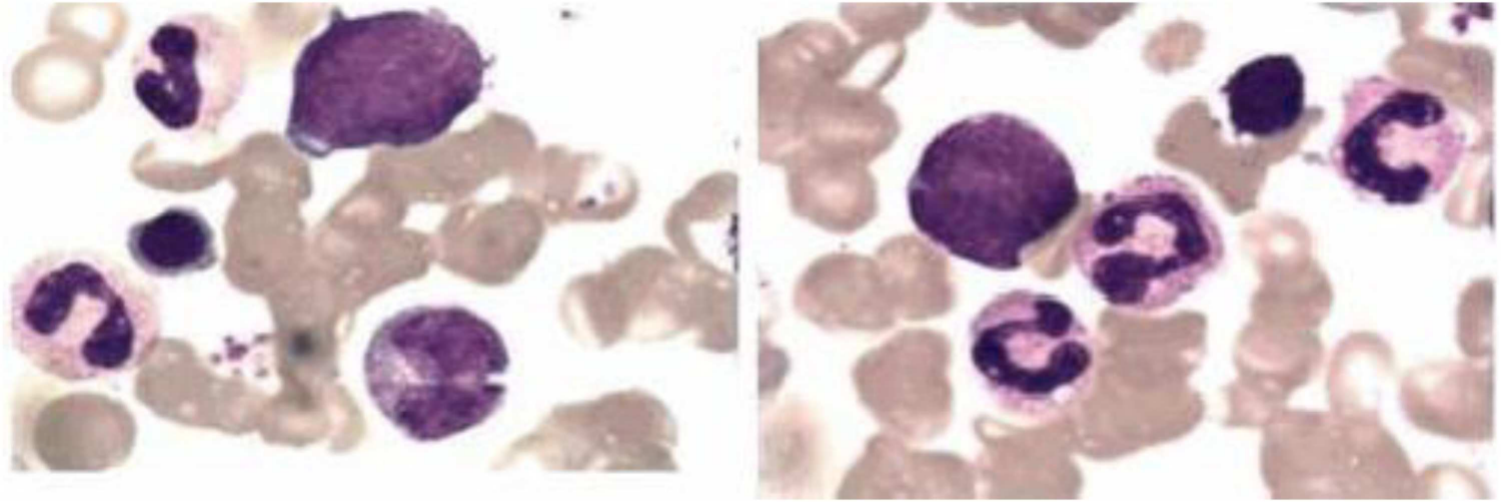
The bone marrow analysis of pediatric patients with majeed syndrome reveals that erythroid cells constitute approximately 21% of the total bone marrow cellular composition, predominantly comprising intermediate and late-stage erythroblasts. Additionally, the mature erythrocytes exhibit anisocytosis.

Extensive infectious workup was negative, including anti-streptolysin O, Epstein–Barr virus, respiratory pathogens, parvovirus B19, and Mycoplasma pneumoniae. Imaging of the lower limbs revealed bone marrow edema and soft tissue swelling involving both distal femurs (Figure 1), the proximal left tibia, and both ankles. Chest CT showed enhanced lung markings, while abdominal and pelvic CT revealed pneumatosis of the stomach and colon. Bone marrow cytology showed 21% erythroid precursors (Figure 2), predominantly at the intermediate and late stages, with mild anisocytosis among mature erythrocytes.

Given the incomplete response to JIA-directed therapy and the presence of anemia and osteomyelitis-like imaging changes, a monogenic autoinflammatory disease was suspected. Genetic testing was therefore performed with parental consent. Peripheral blood (2 ml) was collected from the patient and her parents for whole-exome sequencing, and candidate variants were confirmed by Sanger sequencing and interpreted using ACMG guidelines. In addition, RNA samples were collected in PAXgene tubes, reverse-transcribed into cDNA, and analyzed by gel electrophoresis and sequencing.

**Figure 3:**
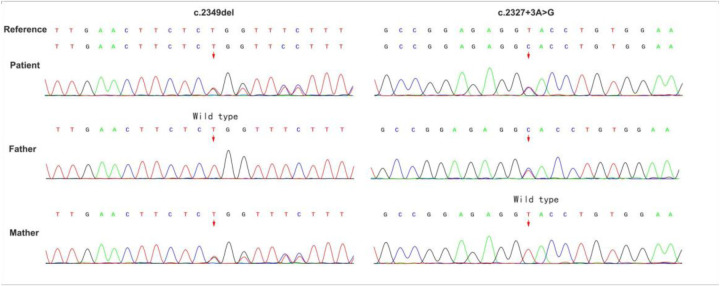
Sequencing diagram of LPIN2 gene mutations in the child and her parents. The arrow indicates the mutation site.

**Figure 4:**
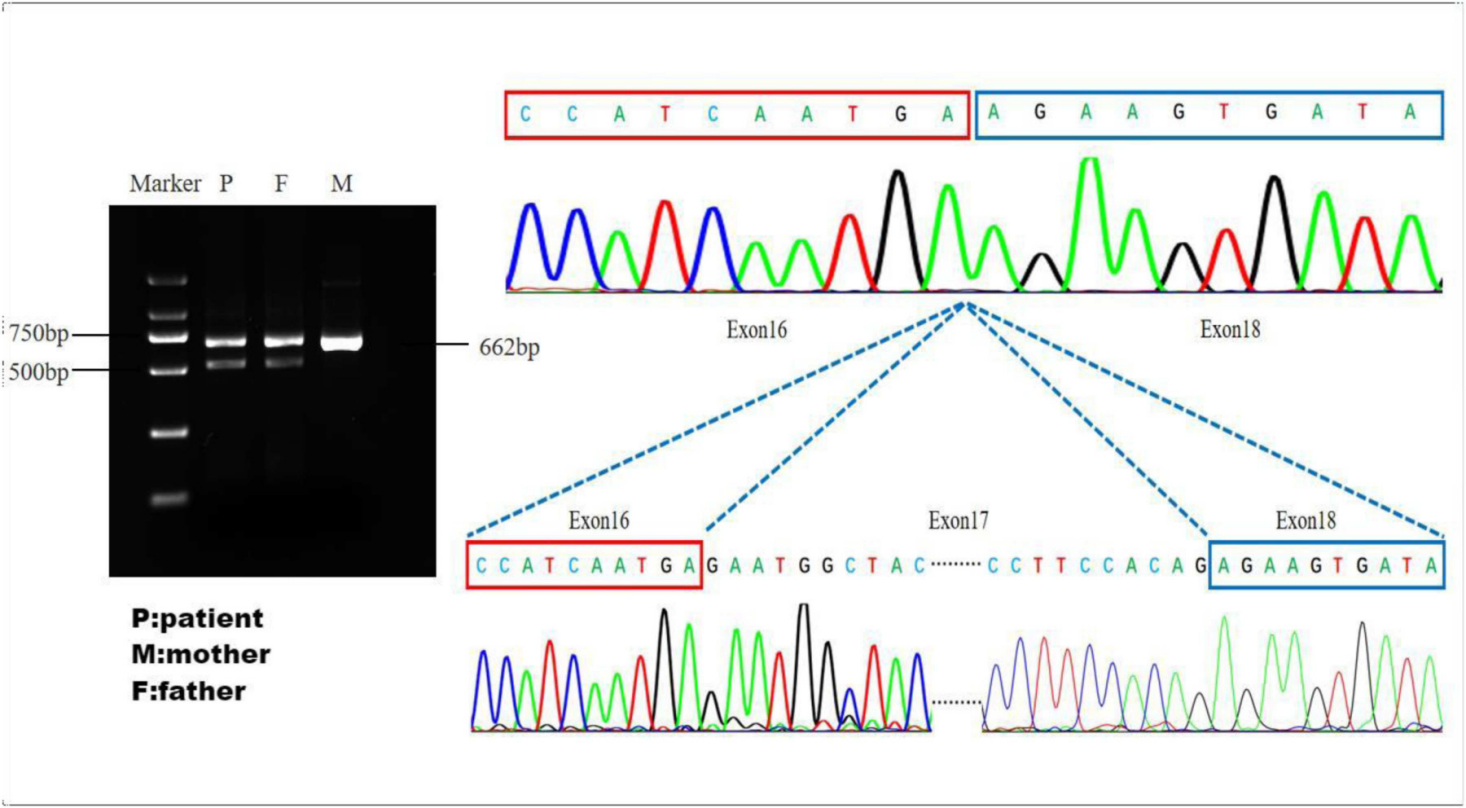
Validation of splice site function: the father and the patient have abnormal cutting bands. The sequencing plot shows that abnormal splicing has caused a skipping of exon 17.

**Figure 5:**
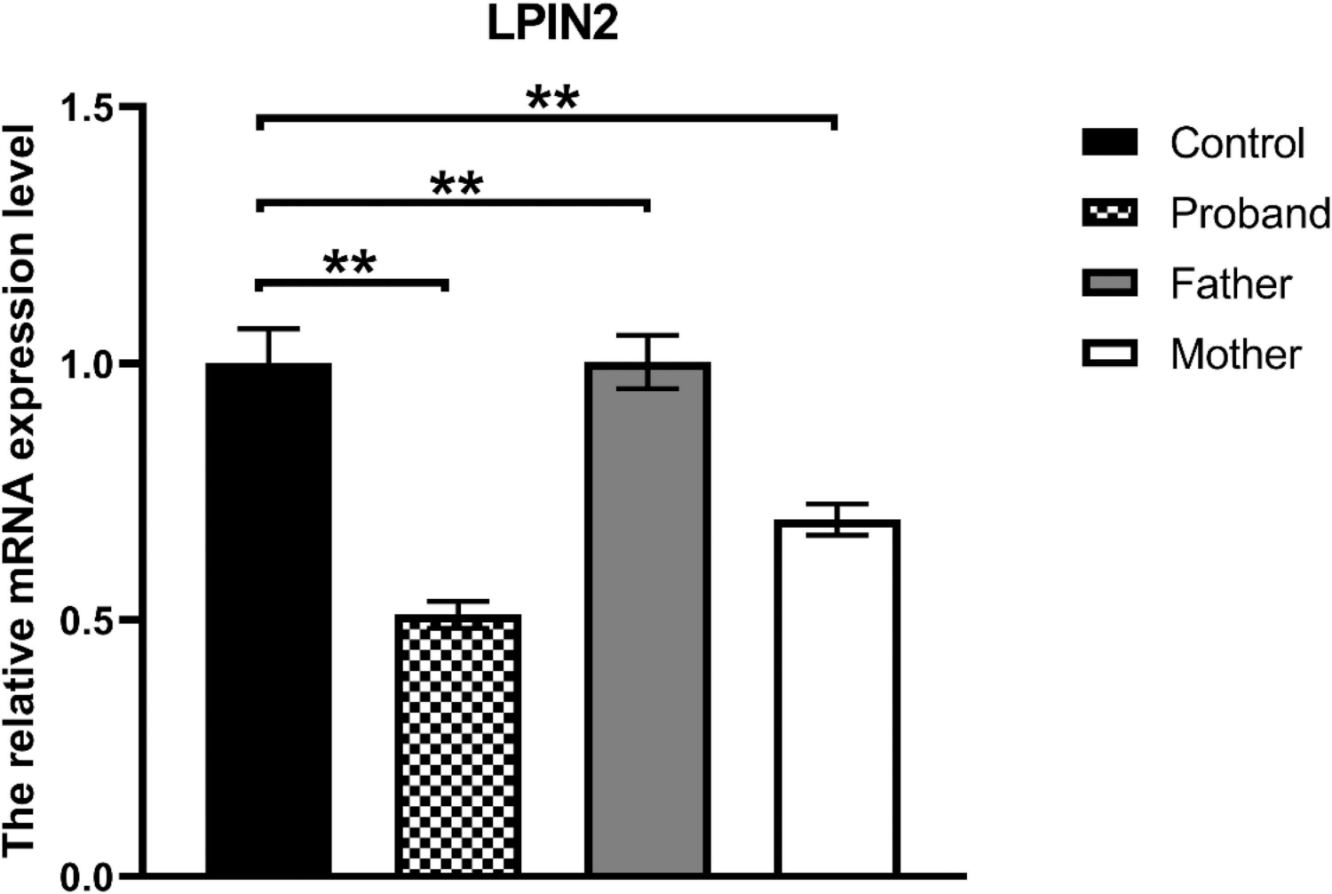
Quantitative RT-PCR: the LPIN2 mRNA level in the proband's father was within the normal range. In contrast, both the proband and the mother exhibited approximately half the normal level of LPIN2 transcript.

Whole-exome sequencing revealed a novel compound heterozygous mutation in LPIN2: c.2349del (p.Glu784ArgfsTer8), inherited from the mother, and c.2327+3A>G, inherited from the father (Figure 3). The first variant was classified as likely pathogenic, while the second was predicted by VarSEAK to alter mRNA splicing. Neither variant was reported in HGMD, dbSNP, or the 1000 Genomes Project. RNA analysis confirmed abnormal splicing with exon 17 skipping (Figure 4), carried out quantitative RT-PCR analysis of LPIN2 mRNA, quantitative RT-PCR analysis revealed that the LPIN2 mRNA level in the proband's father was within the normal range. In contrast, both the proband and the mother exhibited approximately half the normal level of LPIN2 transcript (Figure 5). Taken together with the clinical features of recurrent multifocal osteomyelitis and congenital dyserythropoietic anemia, these findings established the diagnosis of Majeed syndrome.

To contextualize our findings, we conducted a literature review of Majeed syndrome cases published in Wanfang, CNKI, Chinese Medical Journal, and PubMed up to December 31, 2023, using the keywords “Majeed syndrome” and “LPIN2.” After excluding reports without sufficient clinical or genetic data, 35 patients were identified (Table 1). The majority of cases originated from the Middle East and South Asia, reflecting the higher prevalence of consanguineous marriages in these regions. The age of onset was predominantly within the first year of life, although later-onset cases up to 26 months were also documented. Clinically, nearly all patients presented with chronic recurrent multifocal osteomyelitis (CRMO), while congenital dyserythropoietic anemia (CDA) was variably reported. Recurrent fever and joint swelling were frequent manifestations, and Sweet syndrome was observed in a minority. Laboratory findings consistently demonstrated anemia, elevated ESR and CRP, and variable microcytic indices. At the molecular level, diverse pathogenic variants in LPIN2 were identified, including frameshift, missense, and splicing mutations. Several recurrent hotspots, such as c.2201C>T (p.Ser734Leu) and c.540_541delAT, were noted, but novel variants continue to expand the mutational spectrum. Treatments included NSAIDs, corticosteroids, bisphosphonates, methotrexate, and, more recently, biologics such as anakinra and canakinumab, with IL-1 blockade providing the most consistent benefit. Collectively, these reports highlight the heterogeneity of Majeed syndrome (Table 2) and underscore the importance of genetic confirmation to guide targeted therapy.

**Table 1:** Clinical manifestations and molecular characteristics of 35 patients with Majeed syndrome.

Case	Author (Year)	Region	Sex	Age at onset	Recurrent fever	Failure to thrive	CRMO	CDA	Joint swelling	Sweet syndrome	Hb (g/L)	MCV (fL)	ESR (mm/h)	CRP (mg/L)	LPIN2 mutation	Treatment
A1	Majeed et al. (1,989)	Arabic	M	1 mo	+	+	+	+	NR	+	77	65.5	80–100	NR	c.2,201C>T (p.Ser734Leu)	NSAIDs, CS, COL, BT
A2	Majeed et al. (1,989)	Arabic	F	10 mo	+	+	+	+	+	+	52–79	NR	80–100	NR	c.2201C>T (p.Ser734Leu)	NSAIDs, CS, COL, BT
A3	Majeed et al. (1989)	Arabic	F	9 mo	+	+	+	+	+	+	52–80	NR	80–100	NR	c.2201C>T (p.Ser735Leu)	NSAIDs, CS, COL, BT
A4	Majeed et al. (1989)	Arabic	M	9 mo	+	+	+	+	+	+	71–105	59–68	80–100	NR	c.2201C>T (p.Ser736Leu)	NSAIDs, CS, COL, BT
B1	Majeed et al. (2001)	Arabic	F	6 mo	+	+	+	+	+	+	60	70	68	NR	c.540_541delAT	NSAIDs, CS, BT
B2	Majeed et al. (2001)	Arabic	M	2 mo	+	+	+	+	+	+	40	60	127	NR	c.540_541delAT	NSAIDs, CS, BT
C1	Mosawi et al. (2007)	Arabic	F	15 mo	+	NR	+	+	+	–	75	67.8	96	30	R776Sfs*66	NSAIDs, CS
D1	Herlin et al. (2012)	Türkiye	M	3 mo	–	NR	+	–	–	NR	90	NR	96	23.7	c.1316_1317delCT (p.Ser439Trpfs*15)	Canakinumab
D2	Herlin et al. (2012)	Türkiye	M	6 mo	+	NR	+	–	+	NR	97	NR	92	19.6	c.1316_1317delCT (p.Ser439Trpfs*15)	Canakinumab
E1	Rao et al. (2015)	India	M	2 y	–	+	+	+	+	NR	79–99	64.6–71.4	53–140	NR	c.2241_2243delinsGG	MTX, PAM
E2	Rao et al. (2015)	India	M	8 y	–	–	+	+	+	NR	112	86.8	45	NR	c.2241_2243delinsGG	MTX, PAM
F1	Ferguson et al. (2016)	Jordan	F	NR	+	+	+	+	NR	NR	NR	NR	NR	NR	c.2201C>T (p.Ser734Leu)	NR
F2	Ferguson et al. (2016)	Jordan	M	NR	+	+	+	+	NR	NR	NR	NR	NR	NR	c.2201C>T (p.Ser734Leu)	NR
F3	Ferguson et al. (2016)	Jordan	M	NR	+	+	+	+	NR	+	NR	NR	NR	NR	c.2201C>T (p.Ser734Leu)	NR
F4	Ferguson et al. (2016)	Jordan	M	NR	+	+	+	+	NR	+	NR	NR	NR	NR	c.2201C>T (p.Ser734Leu)	NR
G1	Ferguson et al. (2016)	Jordan	M	NR	+	+	+	+	NR	NR	NR	NR	NR	NR	c.540_541delAT (p.Cys181*)	NR
G2	Ferguson et al. (2016)	Jordan	F	NR	+	+	+	+	NR	NR	NR	NR	NR	NR	c.540_541delAT (p.Cys181*)	NR
H1	Moussa et al. (2017)	Spain	M	6 y	–	+	+	NR	+	NR	NR	NR	70	59	c.2327+1G>C	Anakinra
I1	Zakiya et al. (2018)	Bahrain	M	11 mo	+	–	+	+	+	NR	93	NR	50	70	c.2327 + 1G>C	Anakinra
I2	Zakiya et al. (2018)	Bahrain	F	17 mo	NR	–	+	+	+	NR	77	NR	NR	54	c.2327+1G>C	Anakinra
J1	Roy et al. (2019)	Pakistan	F	NR	+	+	+	+	NR	NR	NR	NR	90	110	c.2208G>A (p.R736H)	NR
J2	Roy et al. (2019)	Pakistan	M	NR	–	–	+	+	NR	NR	NR	NR	NR	NR	c.2208G>A (p.R736H)	NR
J3	Roy et al. (2019)	Pakistan	M	NR	–	–	+	+	NR	NR	NR	NR	NR	NR	c.2208G>A (p.R736H)	NR
K1	Liu et al. (2020)	China	M	6 mo	+	NR	–	+	NR	NR	85–95	NR	79	39	c.2327+1G>C; c.1691_1694delGAGA	None
L1	Bhuyan et al. (2021)	USA	F	26 mo	–	–	+	+	NR	NR	101	65.2	77	104	c.1550G>A (p.R517H)	Canakinumab
M1	Pallavi et al. (2022)	India	M	8 d	+	–	+	+	+	+	83–112	63.9–75.7	25–105	310–980	c.2206C>T(p.Arg736Cys)	ALN, MTX
M2	Pallavi et al. (2022)	India	M	7 d	+	+	+	+	+	NR	56–61	56.6–59	36–105	Positive	c.2041delT (p.Trp681Glyfs*14)	ALN
M3	Pallavi et al. (2022)	India	F	8 mo	–	+	+	+	+	NR	61–107	45.6–62.8	30–145	ND	c.2174+4_2174+5del	MTX
M4	Pallavi et al. (2022)	India	M	8 mo	–	+	+	+	+	+	90–126	60.2–78.4	10–108	24.6–67	c.1961G>A (p.Gly654Asp)	ALN
M5	Pallavi et al. (2022)	India	M	2 mo	+	+	+	+	+	NR	70–108	55–82	16–96	81–134	c.1620+5G>C	AZA, SSZ, Etanercept, Adalimumab
N1	Vaishnavi et al. (2023)	India	M	4 y	–	–	+	+	–	NR	90	NR	115	55	c.2207G>A (p.Arg736His)	NSAIDs, ALN, TOFA
N2	Vaishnavi et al. (2023)	India	F	1 y	+	+	+	+	+	+	88	NR	110	59	c.1157C>G (p.Ser386Ter)	NSAIDs, ALN, TOFA
O1	Merve et al. (2023)	Syrian	F	30 y	+	–	+	–	+	NR	74	78.8	104	145	c.1691_1694delGAGA (p.R564fs*3)	Anakinra
P1	Muserref et al. (2023)	Türkiye	M	18 mo	NR	NR	+	–	–	NR	111	NR	50	3	c.589C>T	Anakinra
P2	Muserref et al. (2023)	Türkiye	F	12 mo	NR	NR	–	–	+	NR	86	NR	120	14	c.589C>T	Anakinra

Hb, hemoglobin; MCV, mean corpuscular volume; CRMO, chronic recurrent multifocal osteomyelitis; CDA, congenital dyserythropoietic anemia; CS, corticosteroids; MTX, methotrexate; NSAIDs, non-steroidal anti-inflammatory drugs; COL, colchicine; PAM, pamidronate; ALN, alendronate; TOFA, tofacitinib; AZA, azathioprine; SSZ, sulfasalazine; NR, not reported; +, present; –, absent.

**Table 2:** Summary of clinical characteristics and treatment patterns in 35 reported patients with majeed syndrome.

Category	Frequency (*n*/*N*)	Percentage (%)
Gender		
Male	23/35	65.7
Age at onset		
≤3 years	22/26	84.6
Clinical features		
Recurrent fever	23/30	76.7
Failure to thrive	20/27	74.1
Joint swelling	19/22	86.4
CRMO present	33/35	94.3
CDA present	29/34	85.3
Elevated inflammatory markers (CRP/ESR/platelets)	23/26	88.5
Treatment received (*n* = 25)		
NSAIDs	9/25	36.0
Methotrexate (MTX)	4/25	16.0
Anti-IL-1 therapy	9/25	36.0
Bisphosphonates (BT)	6/25	24.0
Corticosteroids (CS)	7/25	28.0
Genetic findings		
Heterozygous mutation	5/35	14.3

## Discussion

Majeed syndrome is an exceedingly rare autosomal recessive autoinflammatory disorder caused by biallelic mutations in LPIN2, first reported by Majeed et al. in 1989 (6). It is classically defined by the triad of CRMO, congenital dyserythropoietic anemia (CDA), and, in some cases, neutrophilic dermatoses (2). Since the original description, fewer than 40 cases have been published worldwide, with most reported from regions with high rates of consanguinity. The rarity of this condition and its clinical overlap with more common pediatric diseases, such as juvenile idiopathic arthritis (JIA) and sporadic CRMO, frequently result in diagnostic delays or misclassification.

Our patient was initially treated for JIA because of recurrent arthritis-like symptoms and elevated inflammatory markers. The partial response to NSAIDs, methotrexate, and adalimumab initially reinforced this impression. However, persistent relapses, multifocal bone marrow edema on MRI, and microcytic anemia raised suspicion of a monogenic autoinflammatory syndrome (5). This “diagnostic odyssey” illustrates a frequent pitfall (Table 3): patients with Majeed syndrome are often misclassified as having JIA or sporadic CRMO, leading to prolonged exposure to less effective therapies (2, 7). Literature review confirms that most patients present before the age of three, with recurrent fevers, osteomyelitis, and anemia, and recognizing these red flags—especially early-onset multifocal osteomyelitis combined with unexplained anemia (8)—should lead to prompt consideration of Majeed syndrome and early referral for genetic testing.

**Table 3:** Differential features of majeed syndrome, sporadic chronic recurrent multifocal osteomyelitis (CRMO), and juvenile idiopathic arthritis (JIA).

Feature	Majeed syndrome	Sporadic CRMO	Juvenile Idiopathic Arthritis (JIA)
Etiology	Genetic disorder caused by LPIN2 mutations; autoinflammatory mechanism	Autoinflammatory disease with innate immune dysregulation	Autoimmune disease; exact triggers unknown
Inheritance	Autosomal recessive inheritance confirmed in affected families	Predominantly sporadic; rare familial clustering	Mostly sporadic; some HLA associations
Core Symptoms	Chronic recurrent multifocal osteomyelitis; congenital dyserythropoietic anemia; inflammatory skin lesions	Multifocal sterile osteomyelitis affecting long bones, clavicle, vertebrae	Chronic arthritis persisting ≥6 weeks
Laboratory Findings	Microcytic hypochromic anemia; elevated CRP and ESR	Mild elevation of CRP/ESR; no specific autoantibodies	Elevated CRP/ESR; RF or anti-CCP positivity in subtypes
Imaging	Osteolytic lesions with sclerosis; bone marrow edema on MRI	Lytic lesions with sclerosis and bone marrow edema on MRI	Joint erosions, osteoporosis, joint space narrowing
Histopathology	Neutrophilic sterile osteomyelitis	Chronic inflammation with fibrosis and sclerosis	Synovitis with lymphocytic infiltration
Treatment	Anti-IL-1 therapy (e.g., anakinra) most effective	NSAIDs, TNF inhibitors, bisphosphonates	NSAIDs, DMARDs, biologics (e.g., anti-TNF)

A key novelty of this case is the discovery of two previously unreported LPIN2 variants occurring in compound heterozygosity (9): c.2349del (p.Glu784ArgfsTer8), a frameshift mutation inherited from the mother, and c.2327+3A>G, a splice-site mutation inherited from the father. Neither variant has been reported in HGMD, dbSNP, or the 1000 Genomes Project. Importantly, RNA analysis demonstrated that the splice-site mutation caused skipping of exon 17, quantitative results showed that the skipping in exon 17 caused by the abnormal splicing did not affect the mRNA level of the LPIN2 gene, but exon 17 is located in the C-LIP structural domain, which may disrupt the enzyme's active center and affect its catalytic function. On the other hand, the C-terminus of the LPIN2 protein contains important nuclear localization signals, and the protein produced by the deletion of this exon is likely unable to enter the nucleus properly to perform its regulatory function in the nucleus.This finding is notable because functional validation of splice-altering variants has rarely been documented in published cases of Majeed syndrome.

The identification of these variants not only broadens the known mutational spectrum of LPIN2 but also provides insight into potential genotype–phenotype correlations. In our patient, the hematological manifestations were relatively mild, with microcytic anemia that did not necessitate transfusion. A plausible explanation is that the compound heterozygous state, which includes a splice-site mutation, may permit some residual Lipin-2 activity (10), thereby attenuating disease severity. This interpretation is consistent with previous reports suggesting that patients carrying missense or splice-site variants often exhibit milder anemia than those with biallelic frameshift or nonsense mutations. Nevertheless, additional functional studies will be needed to validate this hypothesis.

Among 35 previously reported cases (Tables 1, 2), 94.3% had CRMO, 85.3% had CDA, and 76.7% had recurrent fever. The male-to-female ratio was approximately 2:1, and 84.6% of patients presented before the age of three. Notably, Sweet syndrome and other neutrophilic dermatoses were rare but distinctive features in some cohorts. Our case is consistent with the typical phenotype of early-onset CRMO and recurrent fever but adds to the growing recognition of clinical heterogeneity, particularly regarding CDA severity.

Conventional therapies such as NSAIDs, corticosteroids, and methotrexate may alleviate symptoms temporarily but fail to address the underlying IL-1-mediated inflammatory process (11). In contrast, IL-1 blockade has shown dramatic efficacy (12). Herlin et al. documented complete resolution of osteomyelitis lesions with anakinra (13), and subsequent studies have confirmed sustained remission with canakinumab and other IL-1 antagonists (14, 15). The incomplete response of our patient to JIA-directed treatments underscores the value of establishing an accurate genetic diagnosis early, as this directly guides the decision to introduce IL-1 inhibitors. Broader access to genetic testing for children with recurrent multifocal osteomyelitis or JIA-like features is therefore critical, serving not only as a diagnostic tool but also as a therapeutic turning point.

Emerging mechanistic studies further support IL-1 as the central driver. Bhuyan et al. demonstrated that LPIN2 mutations lead to dysregulated M2 macrophage function and enhanced osteoclastogenesis, linking inflammation to bone pathology (4). These findings open the possibility of novel adjunct therapies targeting macrophage polarization or osteoclast activity for patients with incomplete response to IL-1 blockade.

This report contributes several unique aspects to the existing literature. First, it documents a novel compound heterozygous mutation in LPIN2, with functional confirmation of aberrant splicing. Second, it illustrates the diagnostic challenge of differentiating Majeed syndrome from JIA, underscoring the necessity of early genetic testing in children with refractory arthritis-like symptoms and anemia. Third, it provides further evidence that CDA severity may vary with specific mutation types, supporting ongoing efforts to refine genotype–phenotype correlations. Finally, it reinforces that early institution of targeted IL-1 blockade could dramatically alter disease trajectory, reducing morbidity and preventing long-term complications.

## Conclusion

In conclusion, our case of Majeed syndrome caused by a novel compound heterozygous LPIN2 mutation expands the known mutational landscape of this rare disease and emphasizes the clinical importance of early recognition. For pediatric patients with early-onset multifocal osteomyelitis and anemia, particularly when refractory to JIA therapies, genetic testing are recomments as timely diagnosis not only provides clarity but also enables the use of targeted IL-1 inhibitors, which remain the most effective treatment strategy identified to date.

## Data Availability

The original contributions presented in the study are included in the article/Supplementary Material, further inquiries can be directed to the corresponding authors.
